# Hepatitis C in healthcare personnel: follow-up analysis of treatments with direct-acting antiviral agents

**DOI:** 10.1186/s12995-021-00320-4

**Published:** 2021-08-24

**Authors:** Claudia Westermann, Dana Wendeler, Albert Nienhaus

**Affiliations:** 1 Competence Centre for Epidemiology and Health Services Research for Healthcare Professionals (CVcare), Institute for Health Services Research in Dermatology and Nursing (IVDP), University Medical Centre Hamburg-Eppendorf (UKE), Martinistr. 52, 20146 Hamburg, Germany; 2 Department for Occupational Medicine, Hazardous Substances and Public Health (AGG), German Social Accident Insurance, Institution for the Health and Welfare Services (BGW), Pappelallee 33-37, 22089 Hamburg, Germany

**Keywords:** Hepatitis C, Direct-acting antiviral agents, Occupational disease, Healthcare personnel

## Abstract

**Background:**

Hepatitis C infections (HCV) are associated with an increase in morbidity and mortality. The aim of this study is to update the results of treatment with direct-acting antiviral agents (DAAs) using a larger population of healthcare personnel (HP) and a longer observation period.

**Methods:**

Secondary data analysis of DAA treatment administered to HP (with confirmed occupational acquired HCV infection) between 1 January 2014 and 30 December 2018, is based on statutory accident insurance data from Germany. The end points of the study were results of a monitoring carried out 12 and 24 weeks after the end of treatment (sustained virological response, SVR), as well as side effects and the assessment of reduced work ability after treatment. Multivariate logistic regression models were constructed to investigate predictors of SVR.

**Results:**

The study population (*n* = 305) mainly comprised HP with a genotype 1 infection. The average age was 63 (SD 10) and 77% were female. Two thirds of the HP suffered from fibrosis or cirrhosis, and had experience of treatment. Statistically, men were significantly more likely to suffer from cirrhosis than women (60% compared to 21%, *p* < 0.001). The end-of-treatment response (ETR) rate was 99% and the SVR12 and SVR24 rates were 98%. Liver cirrhosis proved to be a predictor of a statistically significant reduction in success rates.

**Conclusion:**

DAA treatment leads to high SVR. Early HCV treatment is associated with higher SVR.

**Supplementary Information:**

The online version contains supplementary material available at 10.1186/s12995-021-00320-4.

## Background

Viral hepatitis C (HCV) is one of the most prevalent blood-borne infectious diseases in the world and is often chronic. According to estimates of the World Health Organisation (WHO), around 1% of the world’s population carries a chronic hepatitis C infection (CHC), although only a minority are aware of it [1, 2]. Each year, an estimated 700,000 people die of HCV-related complications such as liver cirrhosis, hepatocellular carcinoma and liver failure. CHC is associated with high morbidity and mortality and there is no vaccine against it [1, 3]. Second-generation direct-acting antiviral agents (DAAs) provide HCV-infected patients with an efficient, orally administered, interferon-free means of treatment, regardless of the extent of liver damage and the genotype [3, 4]. This regimen achieves highly sustained virological responses (SVRs) of over 95% with high compatibility [1]. Although DAA regimens are more effective, they are more costly than interferon-based treatments due to the expensive drugs involved [5]. Healthcare personnel (HP) work in environments with specific accident and disease risks. Contact with HCV-infected patients in invasive activities that entail a greater risk of injury for the employees is significant for occupational exposure [6]. Needle-stick injuries are among the most common occupational accidents reported to the German Social Accident Insurance, Institution for the Health and Welfare Services (BGW) [7]. Despite a decline in figures, HCV infections continue to be among the common infections in healthcare that are recognised as occupational diseases and form the basis for approval of pensions at BGW [7]. The analysis of BGW’s data shows a continuous decline in incidence rates of occupational HCV infections with a significant increase in costs. These costs are explained by the increase in compensation payments for occupational pensions and by a rise in the costs for drugs since the introduction of DAAs [8]. This follow-up study aims to update the treatment results for the new DAA therapies among HP as well as the effects of these on the reduced work ability (RWA) using a larger population and a longer observation period.

## Methods

### Data sources

This is a secondary data analysis of second-generation DAA-based therapies among HP with an HCV infection confirmed as an occupational disease. The update was conducted using routine data from BGW. It is based on the initial analysis of these therapies and follows the methodology and analysis strategy of that analysis [9]. The analysis included anonymised data from insured HP who received DAA treatment between 1 January 2014 and 30 December 2018 and for whom results were available 12 weeks after the end of treatment. Treatment failure immediately after the end of treatment was also assumed in the absence of data at 12 or 24 weeks after the end of therapy. These cases were included in the analysis as well. Data was provided on gender, age, genotype, RWA, treatment administered, treatment status (naive/experience), cirrhosis (yes/no), duration of treatment, treatment results (evidence of RNA), side effects. In accordance with the Professional Code for Physicians in Hamburg (Art. 15, 1) and the Chamber Legislation for Medical Professions in the Federal State of Hamburg (HmbKGH) the analysis of anonymized data is exempt from obtaining advice on questions of professional ethics and professional conduct from an Ethics Committee.

### Assessment of RWA in procedures for occupational diseases

In accordance with the 7th Book of the German Social Security Code (SGB VII), every employer is required by law to insure employees against accidents at work. Benefits from the statutory accident insurance scheme apply to insured persons who are injured or suffer from an occupational disease following an accident at work. They are entitled to compensation if their ability to perform and thus their ability to work cannot be fully restored. This entitlement to an injured person’s pension depends on the assessment of the RWA and on the extent to which the reduction in an insured person’s physical and mental capacity restricts his or her ability to work. In the event of complete loss of capacity to work (100%), a full pension is paid, which amounts to two thirds of the annual earnings before the occupational disease. In case of partial RWA, a partial pension is paid according to the degree of RWA. The entitlement begins with an RWA of at least 20%. For beneficiaries suffering from HCV, the RWA depends on the fibrosis stage and the degree of inflammatory activity of the disease. Grading, however, should be done on an individual basis and personal circumstances such as fatigue or depression should also be taken into account.

### End points and statistical analyses

The end points of the study were results monitored 12 and 24 weeks after the end of treatment (SVR12 and SVR24), documented side effects and the results of assessment of the RWA after the conclusion of DAA treatment. If data were missing at 12 or 24 weeks after the end of therapy and treatment failure occurred immediately after DAA treatment, treatment failure was also assumed. Evidence of viral RNA after prior SVR was considered to be a relapse. Descriptive statistics (absolute number (frequency) and mean values with standard deviation (SD)) were provided. Crosstabs were carried out to investigate associations between treatment success (SVR12) and treatment status (naive/experienced), cirrhosis (yes/no), RWA (< 50%/≥ 50%) and gender using Fisher‘s exact test. Factors of influence for the SVR12 target value underwent univariate and multivariate analysis using logistical regression with odd ratios (OR) and 95% confidence intervals (95% CI) being specified. The Pearson correlation coefficient was used to measure the intensity and direction of the linear relationship between age and RWA and between cirrhosis and RWA ≥ 50%. *P*-values of < 0.05 were deemed statistically significant. Nagelkerke’s R-squared was calculated and used to derive Cohen’s effect size [10]. The data analysis was performed using IBM SPSS Statistics for Windows, version 24.0, Armonk, NY: IBM Corp. (published 2014).

## Results

### Sample description

Over the study period, a total of 306 healthcare employees received treatment with a DAA regimen. Data on SVR12 was available in 305 treatment cases. The study population (*n* = 305) is described in Table 1. The sample population comprised 77% women. The average age was 63 (SD 10). The insured patients most commonly had a HCV genotype 1 infection; there was no documented co-morbidity with hepatitis B or HIV infections. 71% of the insured patients had a diagnosed liver condition (fibrosis 44%, cirrhosis 27%) prior to DAA therapy, and almost the same proportion had experience of therapy. The majority of insured patients had a pension-relevant RWA of ≥20%, while just under a third had a RWA of ≥50%.

**Table 1:** Baseline characteristics (*n* = 305)

Characteristic	Total	
	n	%
Gender (no missing data)		
Women	234	77
Age (no missing data)		
Mean/SD	63/10	
Median/Minimum/Maximum	63/22/89	
Age group		
≤ 49	22	7
50–59	78	26
60–64	76	25
≥ 65^a^	129	42
Genotype (19 missing data)		
1 Sub-type not recognised	20	7
1a	74	26
1b	161	56
2	10	3
2b	1	< 1
3	14	5
3a	3	1
4	3	1
RWA% (13 missing data)		
0- < 20	22	7
20	110	40
30–40	80	27
50–60	49	16
70–80	14	4
90–100	17	6
Stage of liver disease (51 missing data)		
No findings	73	29
Fibrosis	111	44
Cirrhosis	70	27
Treatment (39 missing data)		
Naive	83	31
Experienced	183	69

^a^ Age upon drawing pension, *RWA* Reduced work ability, *SD* Standard deviation

### Treatment regimens and side effects

The most commonly administered regimen was ledipasvir (LDV) combined with sofosbuvir (SOF) (*n* = 152/50%, Table 2). A combination with ribavirin (RBV) was administered in 54 cases (18%), with (pegylated) interferon (PEG-IFN) and RBV in 3 cases (1%). Therapy mostly lasted 12 weeks (71%) with a range of 6 to 24 weeks. For 66% of insured patients, the treatment was not associated with any side effects. The most common reported side effect was a combination of mild symptoms such as headaches, nausea and fatigue (20%). Just 4% of insured patients reported skin reactions such as pruritus to generalised skin rash and phototoxic reactions. In individual cases, there were concurrent side effects, such as skin reactions or joint and muscle pain, in addition to headaches, nausea and fatigue (*n* = 5, not in Table 2). In rare cases, low haemoglobin counts, feelings of anxiety, irritability and depression as well as gastrointestinal disorders were found. Information from insured patients on other complaints that were not primarily viewed as side effects of the DAA treatment but as the symptoms of an advanced CHC infection (bleeding of the oesophageal varices, incipient hepatorenal syndrome) was summarised under “other side effects”. Low haemoglobin counts occurred in DAA treatments where RBV was combined (*n* = 3). These achieved SVR12 in the regular treatment period (12 or 24 weeks). Anxiety and depression developed where DAA treatment was administered in combination with RBV and/or PEG-IFN (*n* = 4). These too reached SVR12 within expected parameters.

**Table 2:** DAA regimens, results and observed side effects (n = 305)

Characteristic	Total	
	n	%
Treatment (2 missing data)		
SOF, LDV	152	50
SOF, DCV	37	12
SOF, LDV, RBV	25	8
DSV, OBV, PTV, RTV	19	6
DSV, OBV, PTV, RTV, RBV	15	5
SOF, SMV	14	4
GCP, PBV	12	4
SOF, RBV	11	3
SOF, VEL	7	3
EBV, GZP	5	2
SOF, RBV, PEG-IFN	3	2
SOF, DCV, RBV	2	< 2
EBV, GZP, RBV	1	< 1
Treatment results		
Directly after therapy (16 missing data)		
ETR	285	99
Remission/viral load unchanged	4	1
12 weeks after treatment (no missing data)		
SVR12	300	98
Remission/viral load unchanged	1	< 1
Relapse	5	1
24 weeks after treatment (53 missing data)		
SVR24	246	98
Remission/viral load unchanged	5	2
Relapse	0	0
Side effects (1 missing data)		
None	212	70
Headaches, nausea, sleep disorder	60	20
Skin reactions	11	4
Joint and muscle pain	7	2
Depression, anxiety	4	1
Anaemia	3	1
Gastrointestinal disorders	3	1
Other^a^	4	1

^a^ Expression of advanced infection (hepatorenal syndrome *n* = 2, bleeding of the oesophageal varices *n* = 1, kidney failure and pancreatitis *n* = 1), *DAA* Direct-acting antiviral agents, *DCV* Daclatasvir, *DSV* Dasabuvir, *EBV* Elbasvir, *GCP* Glecaprevir, *GZP* Grazoprevir, *LDV* Ledipasvir, *SOF* Sofosbuvir, *OBV* Ombitasvir, *PTV* Paritaprevir, *PBV* Pibrentasvir, *RBV* Ribavirin, *RTV* Ritonavir, *VEL* Velpatasvir, *SMV* Simeprevir, PEG-IFN pegylated interferon, *ETR* End of treatment response, *SVR12* Sustained virological response 12 weeks after end of treatment, *SVR24* Sustained virological response 24 weeks after end of treatment

### Results monitoring after DAA therapy

The end-of-treatment response (ETR) rate was 99% and the SVR12 and SVR24 rates were 98%. Having experience of treatment did not result in any significant difference in a group comparison in relation to treatment results at SVR12 (SVR12 98.9% versus 96.4%, *p* = 0.18). There was a statistically significant difference between the groups in terms of cirrhosis status; insured HP who did not have liver cirrhosis achieved SVR12 more frequently to a statistically significant degree than HP who did have liver cirrhosis (SVR12 99.5% versus 92.9%, *p* = 0.007). For HP with a RWA grading of under 50%, the DAA treatment was also significantly more likely to be successful than for patients with a RWA grading of 50% or higher (SVR12 99.1% versus 95.0%, *p* = 0.05). Women were statistically more likely to reach SVR12 than men (SVR12 99.6% versus 93.0%, *p* = 0.003). Crosstabs results with Fisher’s exact test are presented in the Additional File 1 and were confirmed by univariate logistic regression analysis (Table 3). Differentiation by gender and cirrhosis showed that men had significantly higher rates of cirrhosis compared with women (50.9% versus 20.8%, *p* < 0.001). In the multivariate logistic regression analysis, cirrhosis was confirmed as a predictor for reduced treatment success (Table 3). The presence of liver cirrhosis resulted in a statistically significant reduction in the SVR12 rate with an adjusted OR of 0.05 (95% CI 0.01–0.52; *p* = 0.01). The variables “RWA” and “gender” correlated with the variable “cirrhosis” in the multivariate model and were therefore not included. Age also had an effect on SVR12. With an increase in age, there was also an increase in the probability of therapy success, although this result is not significant (OR 1.06; 95% CI 0.94–1.19, *p* = 0.36). Nagelkerke’s R-squared was 0.18, which corresponds to an effect size of 0.47, which Cohen classifies as large. The liver enzyme laboratory values (GOT, GPT, ɣGT) for 175 HP at 24 weeks after the end of treatment were available at the time of the evaluation. For 153 (87%) of these insured HP, the liver enzymes were in the normal range 24 weeks after the DAA therapy.

**Table 3:** Univariate and multivariate analysis regarding SVR12 (n = 305)

Variable	Missing values	n	OR^**1**^ (95% CI) univariate	***p***-value	OR^**2**^ (95% CI) multivariate	***p***-Value
Cirrhosis	51	254	0.07 (0.01–0.62)	0.02	0.05 (0.01–0.52)	0.01
(no/yes)		(184/70)				
Treatment	39	266	3.39 (0.56-20.71)	0.19	–	–
(naive/experienced)		(83/183)				
RWA	13	292	0.18 (0.03-1.01)	0.05	–	–
(< 50%/≥50%)		(212/80)				
Gender	0	305	0.06 (0.01-0.49)	0.01	–	–
(women/men)		(234/71)				
Age^a^	0	305	0.99 (0.91-1.08)	0.89	1.06 (0.94-1.19)	0.36

^1^ Univariate analysis

^2^ Multivariate analysis with adjusted OR

^a^ as continuous variable

*OR* Odds ratio, *CI* Confidence interval, *SVR12* Sustained virological response 12 weeks after therapy, *RWA* Reduced Work ﻿Ability

### RWA evaluation after DAA treatment

Evaluation of RWA after DAA treatment was done for 247 (81%) of the insured HP, on average 10 months after the end of treatment (Table 4). The RWA was adjusted for 193 (78%) of the HP. The RWA determined before treatment lapsed for 127 HP, 53 had their status decreased after the evaluation and 13 had their status increased. Reasons for an increased status included liver transplant after successful DAA treatment, bleeding of the oesophageal varices, liver cirrhosis decompensation and incipient hepatorenal syndrome. The correlation coefficients between age and RWA (*r* = 0.21; *p* < 0.001) and between cirrhosis and RWA ≥ 50 (*r* = 0.69; *p* < 0.001) were both positive.

**Table 4:** Reduced work ability before and after DAA treatment (*n* = 247)

RWA as %		After DAA therapy						Overall	
Before DAA therapy		0	20	30–40	50–60	70–80	90–100	n	%
	0	15	0	0	0	0	0	15	6
	20	87	7	1	0	0	1	96	39
	30–40	39	14	15	2	0	2	72	29
	50–60	1	4	18	18	1	1	43	18
	70–80	0	0	0	3	4	3	10	4
	90–100	0	0	0	0	4	7	11	4
Overall	n	142	25	34	23	9	14	247	
	%	58	10	14	9	4	5	100	100

*RWA* Reduced work ability, *DAA* Direct-acting antiviral agents

## Discussion

In this study population, HP infected with CHC achieved high SVR12 and SVR24 rates (98%) as a result of the administered DAA regimen. No relapses were observed after a prior SVR12. The presence of cirrhosis proved to be a predictor for reduced treatment success (SVR12). Neither age nor treatment experience had a significant correlation with the target variable SVR12. In the univariate analysis, gender had a statistically significant effect on the success of treatment. Women reached SVR12 more frequently than men. The incidence of liver cirrhosis was significantly higher in men (50.9% versus 20.8%, *p* < 0.001). In the multivariate regression model, cirrhosis was confirmed as a predictor for a reduction in treatment success. This is consistent with international study results [11–13]. The results of a large prospective multi-centre study from France (the ANRS CO22 Hepather cohort) also showed higher SVR rates for patients without cirrhosis compared to patients with cirrhosis (96% versus 92%). According to the authors [12], liver damage and liver inflammation was reduced in patients after DAA therapy where SVR was reached. SVR is associated with liver regeneration, a reduction in the progression risk for liver-related complications and development of hepatocellular carcinoma [12]. SVRs are associated with reducing morbidity and mortality resulting from a CHC infection in CHC patients, irrespective of cirrhosis status, as well as being associated with an improvement in health-related quality of life [12, 14–18]. In the observed study population, positive effects on the patients’ RWA were observed on average just 9 months after successful DAA treatment. An evaluation was carried out in 81% of the insured patients after DAA treatment, showing an improvement in RWA for nearly 80% (*n* = 193 of 247) of those being analysed. Because the increase in the number of pension claims is proportional to the severity of RWA grading, it can be assumed that pension payments will be lower for the observed population in the future. This effect becomes more pronounced the earlier an HCV infection is diagnosed and treated. Taking pension benefits into account, use of DAA treatments in insured HP without cirrhosis correlates with a reduction in costs [5]. Cost-effectiveness models also show that early treatment is more cost-efficient than treatment at a later stage of the disease [15]. However, DAA treatments are still expensive. The optimisation of the treatment duration is a key contributor to reducing the high costs of the DAA regimen with the first approved second-generation DAAs. The combination of the DAA regimen with RBV enabled treatments for patients with liver cirrhosis to be effectively reduced from 24 weeks to 12 weeks in most cases [13]. The predominant treatment duration of 12 weeks in this cohort was comparable with the results of the German Hepatitis Cohort (GECCO) [19]. More recent DAA regimens such as Epclusa (SOF + velpatasvir), Zepatier (elbasvir + grazoprevir) or Maviret (glecaprevir + pibrentasvir) provide cheaper pan-genotypic alternatives today that allow for shorter treatment durations without the need to additionally administer RBV [20–22]. The occurrence of haemolytic anaemia, anxiety and depression with regimens involving combination with RBV or PEG- IFN is documented in the literature [23, 24] and was also observed in the cohort studied here. The highly tolerated DAA regimen pibrentasvir/glecaprevir is an RBV-free/(pegylated) IFN-free alternative with a total SVR12 rate of 98%, a short treatment duration and a high barrier to resistance. It is the only pan-genotypic treatment system for patients with severe to terminal kidney diseases, including dialysis patients, and is suitable for patients after liver transplantation [20, 22]. According to Zeuzem [25], if no HCV RNA is detected 12 weeks after the end of DAA therapy, this is deemed a permanent eradication of the virus. Relapses after this point are rare and are generally due to re-infection. The longer study period enabled us to obtain the results 24 weeks after the end of treatment; all SVR12 results were confirmed. HCV infection therapy requires prior diagnosis. As a result of the non-specific course of the disease, researchers assume that many people around the world may have an HCV infection and are not aware of it [26]. There is no vaccine against HCV infection and prior therapies or a successfully treated infection do not offer protection against re-infection [25, 27].

The case figures presented here do not provide a complete picture for occupational HCV infections in Germany. The BGW only records notifications of occupational illness from employees of non-state institutions. There was no standardised collection of data on co-infection. However, we assume that there is a lower likelihood of co-infection because the study population comprised HP who are regularly examined by in-house doctors. Men are more commonly affected by HIV co-infection and chronic HCV progression than women due to their association with risk groups such as intravenous drug consumers (INC) and men who have sexual intercourse with men (MSM) [26, 27]. Interim results from the GECCO study confirm that men are significantly more likely to have an HCV/HIV co-infection than women [28]. This study also only considered occupational HCV infections. We do not have any information on co-morbidity resulting from factors other than the CHC infection.

## Conclusions

The results of this study show high SVR12 and SVR24 rates (98%) as a result of DAA treatments in HP. The endpoints examined 24 weeks after the end of treatment confirmed the SVR12 results. No relapses were observed in HP who achieved SVR12. These prospective data contribute to the validity of SVR12 results, which are seen as equivalent to cure of infection. HP with CHC and failed past treatments can now be effectively treated. Significant independent predictor of decrease odds of SVR12 was liver cirrhosis. In the study population, positive effects on the HP’s work ability were observed after successful therapy. Further prospective studies are needed for a validated interpretation of treatment results and an assessment of side effects. Successful completion of treatment does not provide protection against re-infection. In the absence of vaccination options, avoidance of needlestick injuries is currently the most important preventive measure.

## Supplementary Information

**Additional file 1.**

## Data Availability

The original data are property of the BGW. The aggregated data as they were provided by the insurance are available upon reasonable request by the head of the department Prof. Dr. Albert Nienhaus (albert.nienhaus@bgw-online.de).

## Abbreviations

BGW: Berufsgenossenschaft für Gesundheitsdienst und Wohlfahrtspflege
CHC: Chronic hepatitis C
DAA: Direct-acting antiviral agent
DSV: Dasabuvir
EBV: Elbasvir
ETR: End-of-treatment sustained virological response rate
GECCO: German Hepatitis Cohort
GCP: Glecaprevir
GZP: Grazoprevir
HCV: Viral hepatitis C
HIV: Human immunodeficiency virus
HP: Healthcare personnel
LDV: Ledipasvir
MSM: Men who have sex with men
PEG-IFN: Pegylated interferon
PBV: Pibrentasvir
PTV: Paritaprevir
OBV: Ombitasvir
OD: Occupational disease
RBV: Ribavirin
RNA: Ribonucleic acid
RTV: Ritonavir
RWA: Reduced work ability
SOF: Sofosbuvir
SVR: Sustained virological response rate
VEL: Velpatasvir
WHO: World Health Organisation
