# Supplementary material for: Hepatitis C in healthcare personnel: follow-up analysis of treatments with direct-acting antiviral agents
Source: J Occup Med Toxicol. 2021 Aug 24;16:34. doi: 10.1186/s12995-021-00320-4 (PMC8383415; doi:10.1186/s12995-021-00320-4)
Supplement: Supplementary file 1 — Additional file 1. [file 12995_2021_320_MOESM1_ESM.docx]

**Additional file 1.** **Table** Univariate analysis with Fisher’s exact test regarding SVR12 (n = 305)

| **Variable** | **Missing values**  **n (%)** | **Categories** | **n** | **SVR12**  **n (%)** | **noSVR12**  **n (%)** | **p-Value** |
| --- | --- | --- | --- | --- | --- | --- |
| Cirrhosis | 51 (16.7) |  | 254 |  |  | 0.007 |
|  |  | no | 184 | 183 (99.5) | 1 (0.5) |  |
|  |  | yes | 70 | 65 (92.9) | 5 (7.1) |  |
| Treatment | 39 (12.8) |  | 266 |  |  | 0.18 |
|  |  | naive | 83 | 80 (96.4) | 3 (3.6) |  |
|  |  | experienced | 183 | 181 (98.9) | 2 (1.1) |  |
| RWA | 13 (4.3) |  | 292 |  |  | 0.05 |
|  |  | <50% | 212 | 210 (99.1) | 2 (0.9) |  |
|  |  | ≥50% | 80 | 76 (95.0) | 4 (5.0) |  |
| Gender | 0 (0.0) |  | 305 |  |  | 0.003 |
|  |  | women | 234 | 233 (99.6) | 1 (0.4) |  |
|  |  | men | 71 | 66 (93.0) | 5 (7.0) |  |

SVR12 sustained virological response 12 weeks after therapy; RWA reduced work ability
